# miR-181c associates with tumor relapse of high grade osteosarcoma

**DOI:** 10.18632/oncotarget.3539

**Published:** 2015-03-12

**Authors:** Mori Federica, Sacconi Andrea, Canu Valeria, Ganci Federica, Novello Mariangela, Anelli Vincenzo, Covello Renato, Ferraresi Virginia, Muti Paola, Biagini Roberto, Blandino Giovanni, Sabrina Strano

**Affiliations:** ^1^ Molecular Chemoprevention Unit, Regina Elena National Cancer Institute, Rome, Italy; ^2^ Translational Oncogenomics, Regina Elena National Cancer Institute, Rome, Italy; ^3^ Department of Pathology, Catholic University, Rome, Italy; ^4^ UOC Radiology, Regina Elena National Cancer Institute, Rome, Italy; ^5^ UOC Department of Pathology, Regina Elena National Cancer Institute, Rome, Italy; ^6^ Medical Oncology A, Regina Elena National Cancer Institute, Rome, Italy; ^7^ Department of Oncology, McMaster University, Hamilton, ON, Canada; ^8^ UOC Orthopedic Surgery, Regina Elena National Cancer Institute, Rome, Italy

**Keywords:** giant cell tumor, osteosarcoma, miRNA profiling, relapse

## Abstract

High-grade osteosarcoma (OS) is characterized by low incidence, high aggressiveness and moderate 5-years survival rate after aggressive poly-chemotherapy and surgery. Here we used miRNA profiling as a tool to possibly predict and monitor OS's development and therapeutic outcome. First, we evaluated the altered expression of selected miRNAs from a case of Giant Cell Tumor (GCT) apparently evolved into an OS. We found that most of modulated miRs were associated with pathways of bone resorption and osteogenesis. miRNA expression also revealed that GCT and OS were distinct tumors. Second, we validated the observed miRNA profile in two independent casuistries of ten GCT (not evolved into malignant tumors) and sixteen OS patients. Interestingly, we found that miR-181c and other three miRNAs identified in the first step of the study were also consistently de-regulated in all OS patients. Ectopic expression of miR-181c reduced cell viability and enhanced chemotherapeutic-induced cell death of U2OS and SAOS2 cells. These findings indicate that: i) miRNAs aberrantly modulated in GCT could be predictive of its development into OS and ii) miRNAs expression could be useful to monitor the OS therapeutic outcome.

## INTRODUCTION

Osteosarcoma (OS) is the most common primary tumor of the bone in young patients. It occurs mainly in the second decade with 60% of patients younger than 20 years old [1]. Since OS occurs very commonly during puberty, a time of rapid bone growth and remodeling, it is likely that factors related to growth and bone development play a role in OS etiology. In particular, during puberty, endogenous sex hormones, growth hormones, and insulin like growth factor1 (IGF1) levels are at their highest, so it is possible that these biological pathways play an important role in tumor development [2, 3]. Osteosarcomas are classified as malignant mesenchymal neoplasms in which the tumor directly produces defective osteoid (immature bone). Almost all conventional OS are high-grade malignant tumours with poor prognosis at the time of inoperable relapse of disease, and 20–25% of the patients have detectable metastases at diagnosis. The 5-year survival rate for patients diagnosed with OS without presence of metastasis is 60–65%, whereas it is only 20–28% for patients with metastasis at diagnosis. Even though the survival rate has improved considerably after the introduction of neo-adjuvant chemotherapy, the need for advances in treatment regimens is still high [4].

Giant cell tumor of bone (GCT) represents around 5% of all primary bone tumors and approximately 20% of benign primary bone tumors. The peak incidence is between 20 and 45 years of age. GCT typically affects the ends of long bones; around 5% affects flat bones, especially those of the pelvis [5]. GCTs are characterized by the presence of large multinucleated osteoclast-like giant cells distributed among mononuclear spindle-like stromal cells and other monocytes [6]. The stromal cells show positive expression of bone markers [7-14]; thereby suggesting a mesenchymal lineage and pre-osteoblast phenotype. *In vitro* and *in vivo* assays demonstrate that the spindle-like stromal cells are actually the neoplastic component of the tumor, favoring the hypothesis of a mesenchymal origin of GCTs [15]. GCTs can rarely transform into a malignant tumor, especially following radiation [16]. However, sporadic cases of OS arising from benign GCT without irradiation in the primary lesion are also reported [17-23].

MicroRNAs (miRNA) are short (17–22 nucleotides) noncoding RNAs that modulate gene expression by inhibition of translation [24]. Recent computational estimations suggest that each miRNA regulates more than 200 target mRNAs, implying that more than one third of protein-coding genes are controlled by miRNAs. miRNAs can regulate multiple processes, including metabolism, proliferation, differentiation, development, and cell death [24], while aberrant miRNAs expression has been associated with oncogenesis and tumor suppressor activity [25]. Recent studies have suggested miRNA implication in skeletal tissue development, like miR-29 for osteoblast phenotype attainment [26] or miR-223 for osteoclast differentiation [27]. Moreover growing evidences propose miRNAs expression as potential biomarkers for the diagnosis and prognoses of different tumors [28]. Here we aimed to use miRNA profile as a tool to predict OS development and therapeutic outcome. The miRNA characterization might be of relevant significance in this disease because many physiopathological characteristics of its initiation and progression are still obscure. In particular, in this work, we started our observation from a case of GCT evolved into an OS where the altered expression of selected miRNAs specifically marked that evolution. Interestingly, most of these miRNAs are endowed with great impact on bone resorption and osteogenesis. Subsequently, we validated that observed signature in a consecutive series of GCT and OS admitted at our Institute. We also found that ectopic expression of miR-181c affected cell viability and enhanced chemotherapeutic-induced cell death of osteosarcoma cell lines.

## RESULTS

### Case presentation

**Fig. 1:**
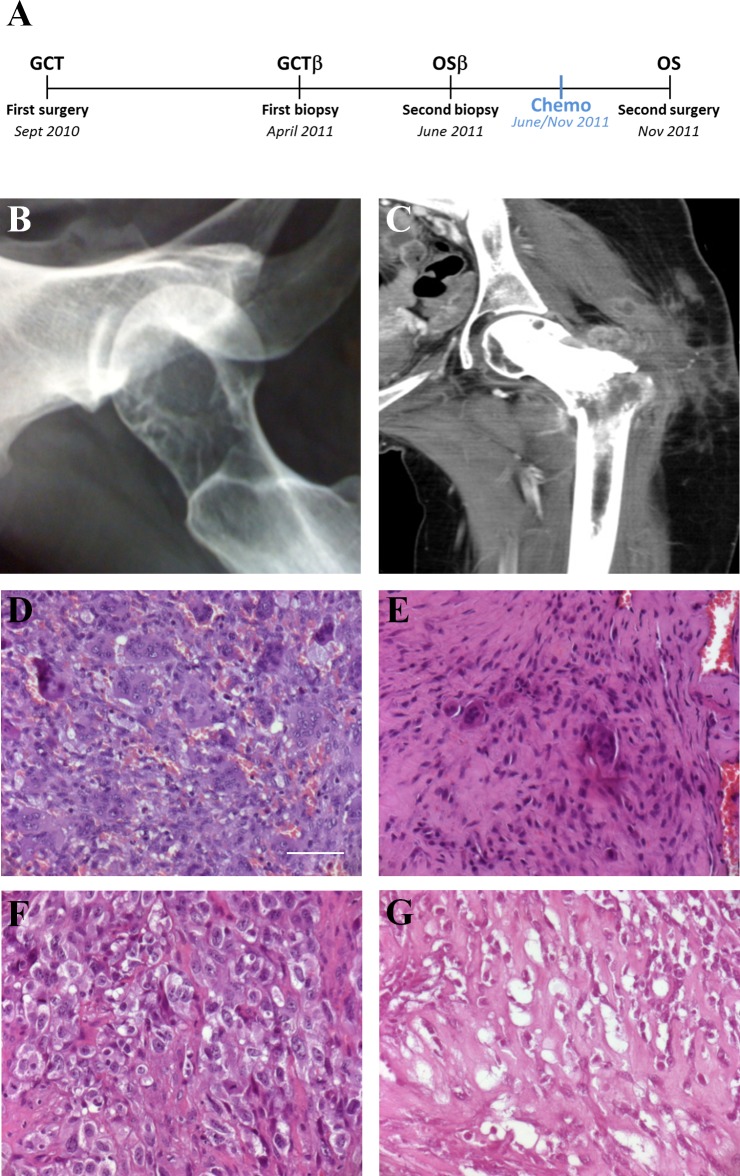
Patient clinical data A. Case Report time-line. B. GCT, X-ray. C. OS local relapse with pathologic fracture, CT D. GCT Haematoxylin/Eosin (H/E) staining: numerous osteoclast-like giant cells scattered among round or spindle mononuclear cells. E: First biopsy (after relapse) H/E staining: mononuclear cells proliferation in fibrous tissue with few giant cells. F: Second biopsy (after relapse) H/E staining: anaplastic mononuclear cells. G: OS H/E staining: intersecting fascicle of pleomorphic cells with osteoid formation. (D-G scale bar: 50μm).

In September 2010 a 22-years-old girl (patient A, whose informed consent has been obtained), with a history of pain on the left hip for approximately 2 months before admission, was referred to Regina Elena National Cancer Institute (IRE) in Rome (Fig. 1A). Plain X-ray revealed an expansive osteolytic lesion in the proximal left femur highly suggestive of GCT (Fig. 1B). A CT-guided needle biopsy was performed afterward; the biopsy tissue showed a lesion composed of numerous osteoclastic giant cells with features identical to stromal cells. There was neither atypia nor atypical mitosis. In consideration of the morphological and radiographic features, a provisional diagnosis of GCT was posed (Fig. 1D). A curettage of the lesion was performed and the histological specimen confirmed the previous GCT diagnosis (Supplementary Fig. 1). On April 2011, 7 months after the surgical treatment, the patient relapsed as confirmed by CT (Fig. 1C) A new biopsy was performed and the GCT diagnosis was confirmed (Fig. 1E). Short time relapse was susceptive of aggressive behavior. Images and subsequent biopsy performed in June 2011, two months after the previous one, confirmed the diagnosis of high grade OS (Fig. 1F). The patient was treated with neo-adjuvant chemotherapy consisting in methotrexate (MTX), Doxorubicin (DOXO) and cisplatin (CDDP) (MAP regimen for 2 courses) and subjected to a hip resection (extra-articular) with a tumor necrosis rate of 65% (Fig. 1G). An adjuvant chemotherapy as for poor responder patients was scheduled but the patient had a quick and dramatic lung progression of disease that led to her death in November 2011.

### miRNA profiling to investigate the clinically observed GCT evolution into OS

**Fig. 2:**
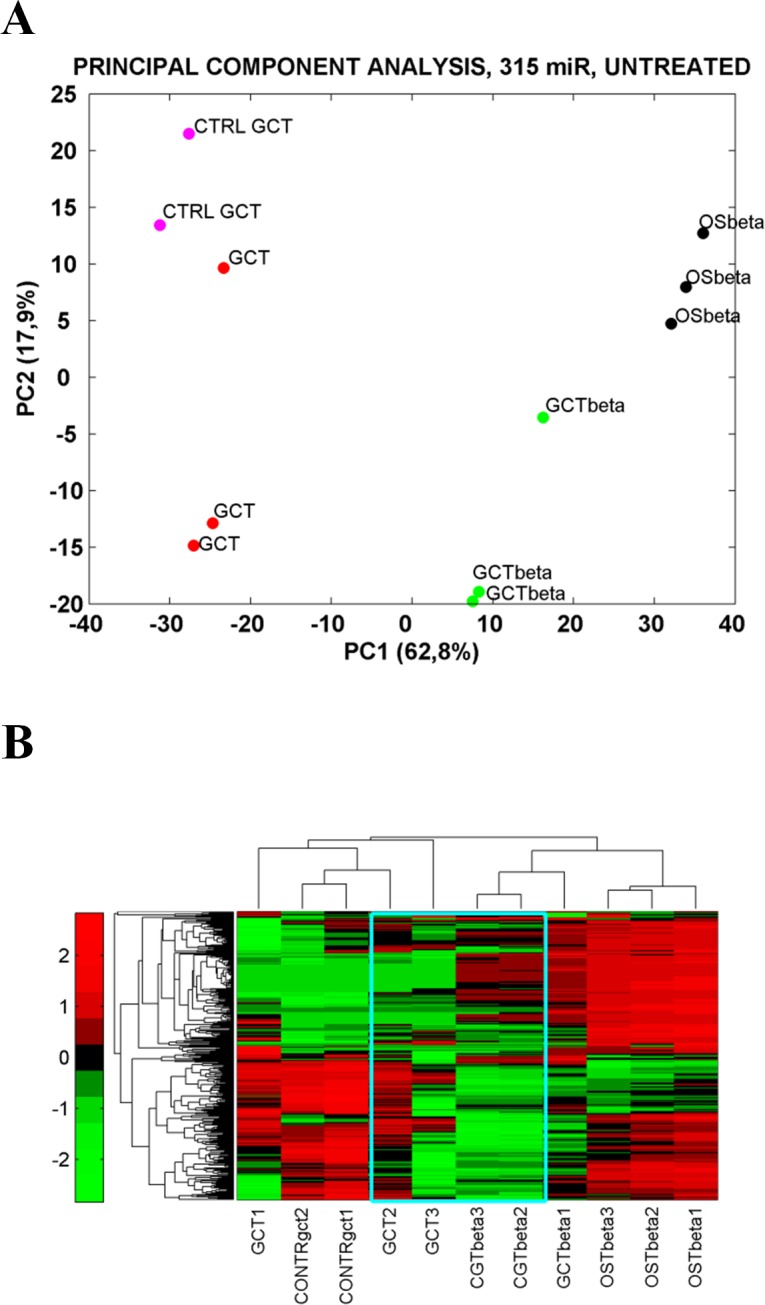
Patient's Giant Cell Tumor and Osteosarcoma molecular profiling A. Unsupervised Principal Component Analysis for expressed miRNAs reveals significant distance among subgroups of samples. In particular, the first component shows a different geometric representation of GCT samples and the subsequent biopsies (GCTbeta, OSbeta). B. Unsupervised two-way Hierarchical clustering for expressed miRNAs grouped samples in clusters of similar expression. Red points have higher expression than mean value while green points indicate a lower expression.

To investigate at molecular level the apparent progression of the GCT towards OS, we assessed the expression of 887 human miRNAs from FFPE samples representing each pathological stage of the studied case (Table 1). We found that 315 miRNAs showed detectable levels of expression. Two independent unsupervised techniques, Principal Component Analysis (PCA) and Hierarchical clustering, were applied to analyze the distribution of the selected 315 miRNAs (Fig. 2). Interestingly, PCA analysis revealed that GCT and its relapse were distinct lesions (Fig. 2A). Considering the PCA first component (PC1), as the data most variance, we observed significant differences between GCT and the subsequent biopsies (Fig. 2A). The analysis of these samples highlighted a specific modulation of selected miRNAs that occurred between the primary GCT and the following relapsed lesions (GCTbeta and OSbeta) (Fig. 2A). Differently, miRNAs expression profile of GCT from patient A showed high correlation with the control sample from the independent patient B (Fig. 2A), corresponding to a primary GCT that did not developed into an OS.

**Table 1:** Case report and control histological samples Patient A (case report) samples were analyzed as triplicate, while Patient B (control) samples were analyzed as duplicate

Derivation	Referred as	Histology	Origin
Surgery	GCT	Giant Cell Tumor	Patient A (case report patient)
I Biopsy	GCTbeta	Giant Cell Tumor	
II Biopsy	OSbeta	Osteosarcoma	
Surgery	CTRL GCT	Giant Cell Tumor	Patient B

Similar results were obtained performing hierarchical clustering. Distinct miRNA expression profiles distinguished GCT and biopsies of the analyzed case report (Fig. 2B). Notably, miRNA expression profile of the GCTbeta, which was a histological sign of GCT relapse, was more similar to OS than to GCT samples from patient A and B.

### Dissection of GCT and OS miRNA expression profiles to identify miRNAs involved in the GCT malignant evolution

**Fig. 3:**
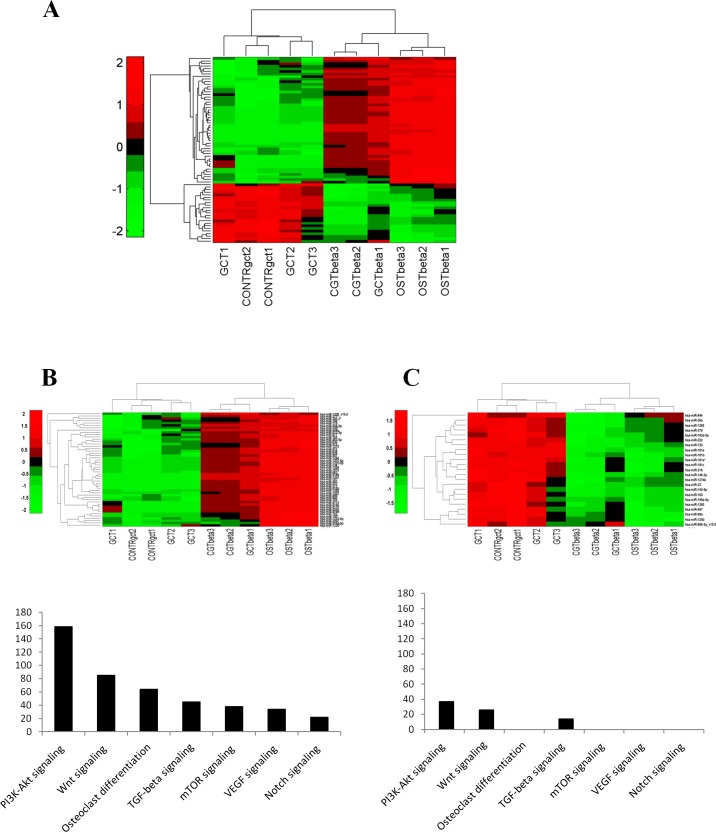
miRNAs deregulated in patient's Giant Cell Tumor vs Osteosarcoma A. Hierarchical Clustering over the selected signature of 70 deregulated miRNAs. B. Particulars of up-regulated and C. down-regulated miRNAs after clustering. All replicates of CGT samples show a different miRNA profile from the subsequent biopsies.

We focused on seventy miRNAs that were differentially expressed between GCT and the subsequent biopsies (Fig. 3A and Supplementary Table 1). To further ascertain the specificity of the modulation, we analyzed by RT-PCR the expression of ten out of seventy miRNAs. Eight of them exhibited a modulation similar to that observed in the array analysis (Table 2).

**Table 2:** miRNAs validation Validation by RT-PCR of eight miRNAs up- or down- regulated in the case report. Results are reported as log_2_ ratio

down-regulated miR	fold (OSbeta\TCG) array	fold (OSbeta\TCG) PCR
miR-378	−2,33	−3,99
miR-181a	−2,89	−3,7486
miR-181c	−3,53	−1,987
miR-193a	−1,1	−1,732
**up-regulated miR**	**fold (OSbeta\TCG) array**	**fold (OSbeta\TCG) PCR**
miR-155	1,29	0,76
miR-198	3,04	2,6197
miR-483-5p	2,37	0,27
miR-630	2,97	0,4908

To investigate the molecular events underlying the modulation of the identified miRNAs we performed an *in silico* prediction of their putative targets. This analysis was performed separately for up- or down-regulated miRNAs comprised in the identified seventy. Interestingly a certain number of putative target genes of up-regulated miRNAs were involved in osteoclast differentiation, Wnt- and TGF-β pathways (Fig. 3B). Considering that miRNAs expression leads to their targets degradation, this suggests a trend toward a suppression of the osteoclast differentiation and, consequently, it could result in an aberrant cellular proliferation. Besides, this effect seems to be strengthened by the down-regulated miRNAs, that targeted these same pathways only marginally (Fig. 3C).

Five miRNAs, miR-193a-5p, miR-181a, miR-181c, miR-378, miR-198 involved in pathways known to be implicated in the OS development, including TGF-β and Wnt, were chosen among the eight validated ones (Table 2) and were further analyzed. miR-193a-5p, miR-181a, miR-181c, miR-378 putative targeted pathways resembled those identified for the seventy miRNAs (Supplementary Table 2).

### Validation of miR-193a-5p, miR-181a, miR-181c, miR-378 down-regulation in OS respect to GCT in two IRE casuistries

To assess whether the modulation of the five miRNAs fished out from the miRNA profiling of the case report could be a feature of OS, we investigated their expression in two distinct casuistries, constituted by ten GCT and sixteen high grade OS patients enrolled at IRE (Table. 3). All the FFPE high-grade OS samples were biopsies from naïve patients, thus comparable with the OS biopsy of the analyzed case report (patient A).

**Table 3:** Osteosarcoma patients' casuistry Features of OS casuistry from Regina Elena National Cancer Institute, Rome. Italy. Therapeutic response and outcome are reported. Tumor relapse score: 0: no relapse. 1: relapse. 2: metastasis

Sample	Age (years)	Gender	BIOPSY	OSTEOSARCOMA's SITE	Tumor relapse	% of necrosis	miR-181c Δct mean
1	17	F	9394/I/2007	proximal tibia dx	1, 2	>90%	5,341442
2	21	M	1390/I/2008	distal femur dx	0	>90%	4,403971
3	35	M	10064/I/2008	femur dx	1	>90%	3,211912
4	12	F	3928/I/2008	distal femur	1	<90%	3,04287
5	63	M	3031/I/2009	omerus sx	2	>90%	4,133101
6	15	M	10600/I/2009	femur dist sx	0	<90%	2,206604
7	25	F	8744/I/2010	wrist dx	2	<90%	4,88121
8	20	M	1102/I/2011	distal femur dx	0	>90%	−2,06748
9	22	F	5355/I/2011	pelvis sx	1, 2	<90%	1,531605
10	11	F	9189/I/2006	distal femur dx	1	<90%	1,417068
11	24	M	5082/I/2007	clavicle dx	2	>90%	6,138674
12	18	M	8688/I/2012	proximal tibia dx	0	<90%	2,729435
13	22	F	5664/I/2012	distal femur sx	0	<90%	2,456497
14	36	M	268/I/2013	distal femur sx	0	>90%	−2,94271
15	18	F	2952/I/2013	proximal tibia sx	0	>90%	−0,07991
16	12	F	724/I/2013	proximal tibia dx	1	<90%	0,969147

**Fig. 4:**
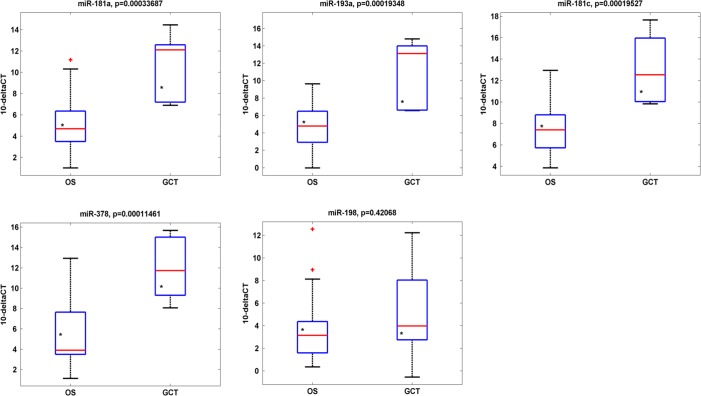
miRNA expression levels in IRE Osteosarcoma casuistry miR-181a, miR193a, miR-181c, miR-378 and miR-198 expression levels in ten GCTs versus sixteen OSs from IRE casuistry. Four out of five miRNAs are down-regulated in GCTs respect to OSs. Asterisks indicate the expression levels relative to case report GCT and case report OS: they are distributed within the values of the GCTs' and OS' samples respectively.

qRT-PCR analysis revealed that four out of five miRNAs identified in the case report analysis, were significantly down-regulated in the analyzed OS casuistry respect to GCT one (Fig. 4) and the patient A GCT and OS (asterisks) are distributed within the values of the GCT's and OS's samples respectively.

### Correlation between miR-181c level of expression in OS patients and therapeutic outcome

**Fig. 5:**
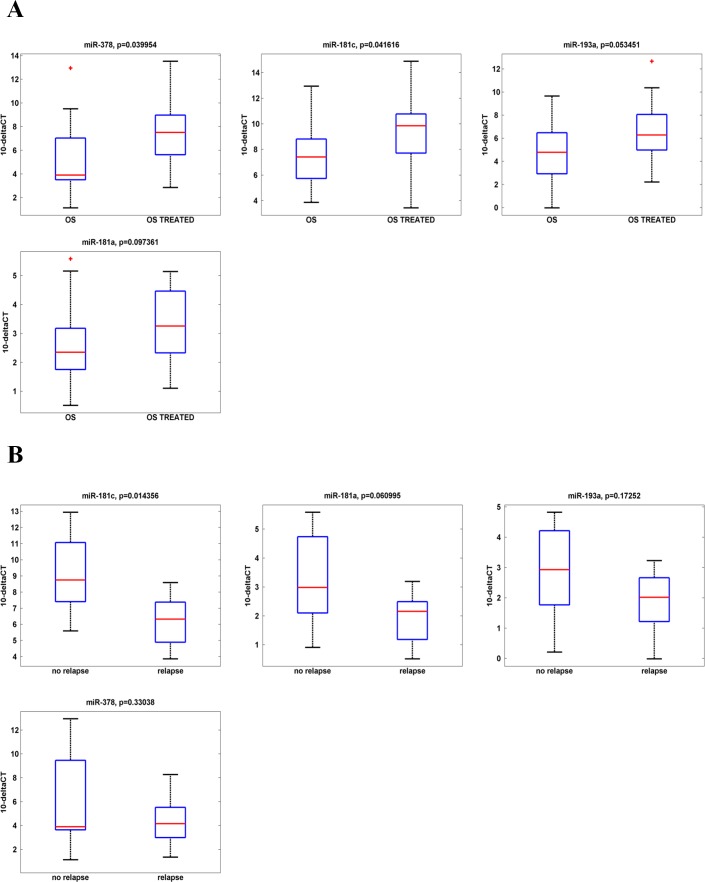
Correlation between miRNA expression levels and patients outcome in IRE Osteosarcoma casuistry A. miR-181a, miR193a, miR-181c and miR-378 expression levels before and after the chemo-therapy treatment. A statistically significant up-regulation in the expression of miR-378 and miR-181c is reported. B. miR-181a, miR193a, miR-181c and miR-378 expression levels in the sixteen OS biopsies from the IRE casuistry. Casuistry samples have been divided between «relapsing» and «not-relapsing». miRNAs expression levels in these samples are reported in the box-plot. A statistically significant correlation is shown between miR-181c down-regulation and the OSs' relapse.

To assess the potential association between the aberrant miRNAs expression and the patients' outcome, we considered two independent parameters: i) the percentage (%) of necrosis induced by the chemotherapeutic treatment and ii) the OS relapse. We first observed that there is apparently no correlation between the % of necrosis induced by chemotherapy and the event of tumor relapse. Indeed, as shown in Table 3, a necrosis index >90% is not always related to the absence of tumor recurrence. We then analyzed the expression of the previously validated four miRNAs levels in the OS biopsies and correlated their values with the necrosis index assessed by the pathologist. As expected, we did not evidence significant predictive correlation between any of the miRNAs expression levels and the efficacy index of the chemotherapeutic treatment (Supplementary Fig. 2). As the necrosis index seemed not to be informative, we then evaluated the effect of the poly-chemotherapy on the expression of the analyzed four miRNAs, in order to find a correlation between the miRNAs expression and the treatment efficacy. From the analysis of miR-193a-5p, miR-181a, miR-181c, miR-378 expression profiles of the sixteen OS biopsies and the matched OS treated samples, we found a statistically significant increment of the expression of miR-181c and miR-378 after the MAP regimen treatment (Fig. 5A).

Then, to assess whether there was an impact of the miRNA up-regulation on the patient outcome, we correlated the expression levels of the analyzed four miRNAs with the patient relapse. As shown in Table 3 the analyzed casuistry included “relapsing” (n=9) and “no relapsing” (n=7) patients. The expression levels of the four miRNAs in the OS treated samples are shown in Fig. 5B. Interestingly, we found that all the four miRNAs levels upregulated by the chemotherapy (Fig. 5A) remain higher in the no-relapsing OS respect to the relapsing ones (Fig. 5B). Indeed, miR-181c down-regulation was statistically significant in “relapsing” tumor patients when compared to those “no relapsing”. An informative trend was found between the other three miRNAs down-regulation and the OSs' relapse. No statistically significant correlation was found between age, gender or histological type and the miR-181c expression (data not shown).

### Ectopic expression of miR-181c affects cell viability of OS cell lines

**Fig. 6:**
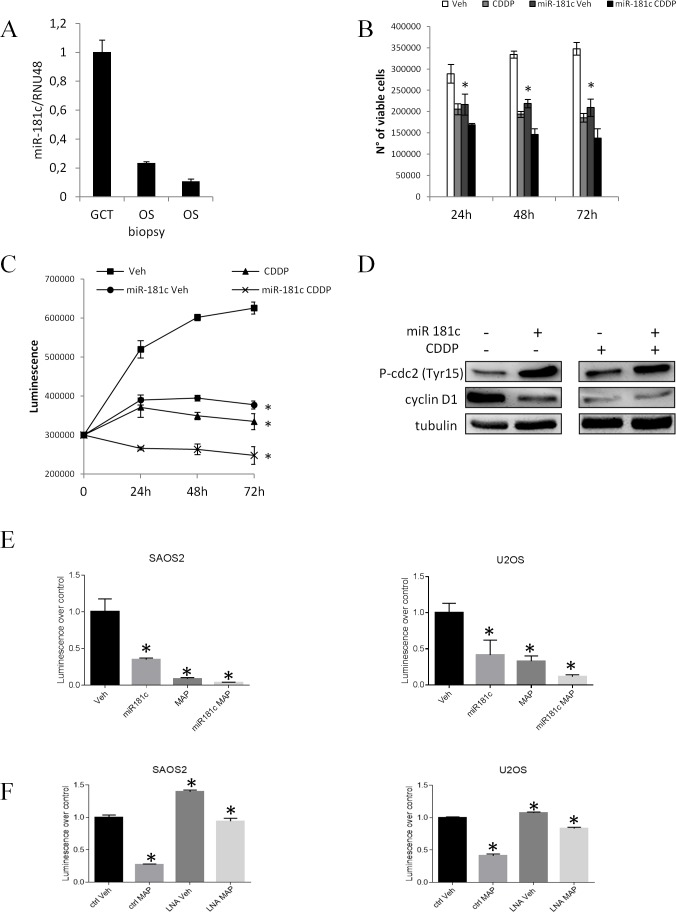
miR-181c overexpression affects osteosarcoma cell viability *in vitro* miR-181c expression levels detected by RT-PCR in the FFPE GCT, OS biopsy and treated OS of the patient A. B n° of viable U2OS cells transfected with control and pCMV-miR-181c vectors after 24h, 48h and 72h from the 5μg/ml CDDP treatment. B. Cell viability measured by ATP-light assay in U2OS cells transfected with control and pCMV-miR-181c vectors after 24h, 48h and 72h from the 5μg/ml CDDP treatment. C. P-Cdc2 incremented and Cyclin D1 reduced expression levels in U2OS cells overexpressing miR-181c and after 24h from the 5μg/ml CDDP treatment. D. ATP-light assay in SAOS 2 and U2OS cells untreated (Veh), treated with MAP poly-chemotherapy regimen (MAP), overexpressing miR-181c (miR-181c) or overexpressing miR-181c and treated with MAP regimen. F ATP-light assay in SAOS 2 and U2OS cells miR-181c inhibited (LNA) or not inhibited (ctrl), untreated (Veh), or treated with MAP poly-chemotherapy regimen (MAP). Data are reported +/− SD. Asterisks indicate statistically significant differences.

From the miRNA expression profiling analysis in the patients' casuistries we found a statistically significant correlation between the miR-181c expression induced by the treatment and the therapeutic outcome. Moreover, quantifying miR-181c concentration in all the tumor phases of the case report (GCT, OS biopsy and treated OS) we revealed a decreasing gradient of expression from the GCT through the relapsing OS (Fig. 6A). To further analyze miR-181c role, we first performed an *in silico* analysis aimed to identify the miR-181c putative targets. As shown in Table 4, we interestingly found that among the others, the pathways affected by miR-181c modulation are the ones of the Cell cycle arrest and of the cell cycle negative regulation. We performed *in vitro* experiments aimed to validate this observation. We analyzed the expression of miR-193a-5p, miR-181a, miR-181c and miR-378 in U2OS osteosarcoma cell line after treatment with 1μg/ml and 5μg/ml cisplatin (CDDP) that represents one of the three drugs administered in the standardized chemotherapy. As for the OS treated patients, CDDP treatment induced miR-181c and the other three miRNA expression levels even in the U2OS cell line (Supplementary Fig. 3A). We ectopically induced miR-181c expression by vector transfection (Supplementary Fig. 3B) and evaluated the effect of miR-181c overexpression induced both ectopically and by CDDP treatment (Supplementary Fig. 3C) on cell viability and cell growth. As shown in Fig. 6, miR-181c overexpression i) resembles CDDP effect on cell viability (Fig. 6B) and cell growth (Fig. 6C) and ii) seems to exert a synergistic effect together with CDDP treatment. Moreover, as CDDP treatment, miR-181c overexpression induced cell cycle arrest, as indicated by the increased level of Cdc2 (Tyr-15) phosphorylation and Cyclin D1 expression reduction (Fig. 6D).

**Table 4:** miR181c putative target genes “Feature” column reported the pathways hosting the miR-181c putative target genes, that are expressed in the “putative targeted genes” column. The pathway analysis has been conducted by the aim of Genemania, on the gene targets identified by DIANA microT.

Feature	FDR	Genes in network	Genes in genome	Putative targeted genes
cell cycle arrest	4.82225E-1	13	192	ATM,NOTCH2,PPM1A,UBC,MAP2K1, ZNF268,CUL5,PSMC2,PHOX2B, GATA6,CAB39,PKD2,ERN1
activation of MAPKK activity	8.90337E-1	6	44	MAP2K1,TNIK,TAOK1,RAP1A, CRK,RAF1
negative regulation of cell cycle	9.036E-1	15	291	ATM,NOTCH2,PPM1A,UBC,MAP2K1, ZNF268,CUL5,PSMC2,PHOX2B,FBX05, GATA6,CAB39,PKD2,ERN1,PTEN
maintenance of location in cell	9.83034E-1	7	91	HSPA5,EPB41L3,ZNF268,CLASP1, GCC2,ANKRD13C,PKD2
1-phosphatidylinositol-3-kinase activity	9.83034E-1	3	11	PIK3R3,PIK3CA,ATM
positive regulation of protein catabolic process	9.83034E-1	6	68	NEDD4,SH3D19,ZNF268,PTEN,VIP, CREBRF
spindle	9.83034E-1	9	141	CLASP1,PKHD1,KIF3A,FBX05,KIF3A, BIRC6,KIFAP3,PKD2,CALM1
regulation of anoikis	9.83034E-1	4	19	PIK3CA,MCL1,ANKRD13C,ITGA5
phosphatidylinositol 3-kinase activity	9.83034E-1	3	12	PIK3R3,PIK3CA,ATM
anoikis	9.83034E-1	4	22	ITGA5,PIK3CA,MCL1,ANKRD13C

To further complement these evidence we then reproduced *in vitro* the MAP chemotherapeutic regimen treating the U2OS and SAOS2 osteosarcoma cell lines, ectopically over-expressing miR-181c, with MTX, DOXO and CDDP. Interestingly cell viability was affected by MAP treatment as well as by miR-181c over-expression and the two conditions revealed an additive effect in the reduction of the cell viability (Fig. 6E). Conversely, inhibition of miR-181c expression by miRCURY LNA™ transfection (Supplementary Fig. 4B), resulted in a slight increase of cell proliferation and in a notably reduced efficacy of MAP treatment (Fig. 6F) Altogether these findings highlight miR-181c down regulation as an alteration that impacts on chemoresistance of OS.

## DISCUSSION

Osteosarcoma is a primary bone malignancy that typically occurs during adolescence but has also a second incidence peak in the elderly. The very low incidence of the disease and its high fatality do not allow methodologically efficient studies and thus many physiopathological characteristics of its initiation and progression are still obscure. There is therefore need of molecular tools endowed with broader diagnostic and prognostic potential. In this report, we describe how specific miRNA modulation may represent molecular biomarkers of OS development. In order to do so, we carefully followed-up a case of GCT ascertained in a young girl which later transformed itself into an OS lesion. McGrath et al., classified GCT which spontaneously evolves into malignant tumors within a short period and without irradiation as “evolutionary” tumors [29]. Twenty-one cases of GCT evolutionary malignancy, with the average period of transformation of 9.9 years have been reported so far. Among those, seven GCT patients progressed into OS [20]. Here we report a case of a GCT evolutionary malignancy that evolved into an OS after an interval of seven months. We found that GCT and OS of the presented case were two distinct tumors considering their miRNA expression. We also found that the first biopsy after relapse, classified as a GCT, exhibited a miRNA expression profile more similar to OS than to that of GCT. These findings might have important implications in the understanding of the malignant evolution of the presented case. First, it might suggest that there were no detectable malignant regions in the primary GCT lesion. Second, some of the differentially expressed miRNAs were already expressed in the first biopsy after relapse, whose histological diagnosis was still GCT. This suggests that early molecular changes at the level of miRNA expression might already occur in apparently benign lesions and drive the progression of GCT to OS. Third, the analysis of the expression of miRNAs can effectively complement and deepen the histological assessment of the presented case.

It is becoming increasingly clear that miRNAs, which can function either as tumor suppressors or oncogenes, feature very accurately the specific stage of a given tumor and can predict its evolution [30, 31]. In addition to this, miRNAs controlling the expression of their mRNA targets can either turn off or on specific pathways whose miRNA-mediated alteration plays a pivotal role in the malignant progression of a tumor. The four miRNAs identified within our profiling analysis have already been published in OSs or in other kind of tumors. miR-181c down-regulation has been shown to be associated with imatinib resistance in chronic myeloid leukemia [32], while in neurodegenerative diseases, glioblastoma and neuroblastoma (NB) studies it has been demonstrated to repress TGFβ1 [33], to attenuate self-renewal ability [34] and to inhibit NB cell growth and metastasis-related traits through the suppression of Smad7 [35] respectively. miR-193a overexpression appears to be correlated with OS patients who were good responders to ifosfamide treatment [36] and miR-378 can function either as an oncogene or a tumor suppressor in different types of cancers. Several studies have reported that miR-378 was significantly down-regulated in Colon Rectal Cancer (CRC). Indeed CRC patients with low miR-378 expression had a significantly poorer overall survival [37]. Consistently with these evidences, we found that these miRNAs were down-regulated in our OS casuistry. As for miR-378, the deregulation of miR-181a expression in cancer development is reported for different types of tumor, even though there is not a univocal variation in its expression. For instance, some studies demonstrated that it is up-regulated in pancreatic cancer and breast cancer [38] [39], and that miR-181a improves proliferation and invasion when ectopically expressed in in OS cell lines [40]. In our study we found that miR-181a is downregulated in OS respect to GCT but we did not find any correlation between its level of expression and the OS ability to relapse. This might suggest that it is not involved in the response to treatment, as also suggested by the *in vitro* experiments (Supplementary Fig. 4A). miR-181a over-expression is also reported in OS [30], but it is important to note that Jones and colleagues detected miR-181a upregulation in OS respect to controls, while we compared miR-181a expression in OSs respect to GCT. Besides, different groups showed that miR-181a was down-regulated in gliomas and aggressive CLL [41] where it functions as a tumor suppressor that triggers growth inhibition, induces apoptosis and inhibits invasion.

*In silico* analysis allowed us to reveal that some of the differentially expressed miRNAs identified in the analysis of the presented case impinge on TGF-β and Wnt-pathways (Fig. 3 and Supplementary Table 2). These two pathways regulate bone resorption and osteogenesis, in particular the Wnt pathway controls osteoblast and osteoclast differentiation and osteosarcoma invasiveness, while the TGF-β pathway stimulates bone and tumor cell proliferation and is associated with high grade osteosarcoma [9, 36, 42-45] [46]. Wnt and TGF-beta inhibition or down regulation, seems to impinge osteosarcoma growth ad metastasis[47] [48] suggesting that Wnt and TGF-beta signaling inhibitors could represent a promising therapeutic strategy. The observed down-regulation of miR-193a-5p, miR-181a and miR-181c is compatible with the engagement of TGF-β- and Wnt- pathways along the GCT evolutionary malignancy of the presented case. Besides, the correlation between the miR-181c overexpression after -and possibly induced by- the MAP treatment and the absence of the tumor relapse, can be partially explained considering that among its putative targets figure genes regulating the cell cycle, like GATA6, MAP2K1, PPM1A and Notch2. To point out some of the already known roles of these genes and their involvement in cancer development, we can mention that the transcription factor GATA6 governs the M-phase of the cell cycle, and has been found to be up-regulated in gastric cancer [49]; MAP2K1 (MEK1) inhibition in combination with BMP-2 and BMP-9 represent a promising strategy in the treatment of the osteosarcoma cells [50]; Notch2 overexpression has been reported in human osteosarcoma biopsy specimens, where the Notch pathway inhibition impinge the osteosarcoma cell proliferation [51] and finally that the PPM1A phosphatase interacts with and dephosphorylates Smad1 [52] leading to its activation that promotes p53 induction to suppress tumorigenesis [53]. Indeed, we observed cell growth arrest in osteosarcoma cell lines as a consequence of the miR-181c overexpression, induced either ectopically or by CDDP treatment.

In line with these findings, a systematic analysis of the miRNA targets and the discovery and functional validation of their related pathways could be a strong rationale to design novel therapeutic approaches to treat OSs. This does not necessarily imply the production of novel therapeutic compounds but could provide the molecular basis for a more tailored use of already existing anticancer drugs. It is worth to notice that we validated miR-181a, miR-181c, miR-193a-5p and miR-378 different modulation between the two casuistries of GCT and high grade OS patients. Besides, the trend we found between miR-181c down-regulation and OS therapeutic outcome, might suggest that the expression of this miRNA could be used to evaluate the treatment response This observation is supported by *in vitro* data, where the overexpression of miR-181c in a human osteosarcoma cell line, correlates with the reduction of cell growth and viability, an effect comparable and synergic with the one exerts by CDDP treatment. These data suggest that miR-181c could be considered a useful indicator in the monitoring of patients therapeutic outcome.

We are aware that the validation in the cohort of sixteen naïve patients is partial and requires further studies on larger cohorts. However it could represent a starting point to i) better and opportunely evaluate GCT relapse when they occur, and ii) help in clarifying OS onset, development and response to therapy.

## METHODS

### Validation samples

The validation of the miRNA signature was done in a consecutive series of ten GCT and sixteen OS patients recruited at the Department of Orthopedic surgery at the Regina Elena National Cancer Institute in Rome. OS patients were aged from 11 to 63, with an average age of 23,2 years. 8 were female and 8 were males. All the tumors were diagnosed and confirmed as high grade OS.

### Features validation

Real-Time PCR (RT-PCR) was used as alternative technique to validate eight out of seventy deregulated miRNAs on case-report samples. We focused our investigation on five out of the eight validated miRNAs, miR-193a-5p and miR-181a, miR-181c, miR-378, miR-198. In particular, miR-193a-5p, miR-181a, miR-181c and miR-378 were involved in the same pathways of the seventy deregulated ones, accordingly to *in silico* putative target prediction analysis (Supplementary Table 2). The RT-PCR of miR-193a-5p and miR-181a, miR-181c, miR-378, miR-198 was conducted on ten GCT samples and sixteen high-grade OS biopsies samples to evaluate fold changes between the two casuistries of lesions.

### miRNA profiling

The miRNA profiling was performed on FFPE sections from samples listed in Table 1 and Table 3. Table 1 reports Patient A specimens from the first (GCT) surgery, as well as from the first (GCTbeta) and second (OSbeta) biopsy after tumor relapse. Another independent patient, corresponding to a primary GCT that did not developed into an OS (Patient B) was included in the analysis. Patient B was added to compare miRNA differently expressed between GCT and OS with their expression in a GCT independent patient. Table 3 reports High-grade OS casuistry collected at Regina Elena National Cancer Institute from 2007 to 2013.

Total RNA was extracted from FFPE samples using miRNeasy FFPE (Qiagen), according to the manufacturer's instructions. Agilent's miRNA Complete Labeling and Hyb Kit (Agilent Technologies Inc., US) was used to generate fluorescent miRNA, following the manufacturer's instructions. Scanning and image analyses were performed using the Agilent DNA Microarray Scanner (P/N G2565BA). Feature Extraction Software (V-10.5) was used for data extraction from raw microarray image files using the miRNA_105_Dec-09FE protocol. Signal intensities were quintile normalization and log2-trasformed. Unsupervised Principal Component Analysis (PCA) and Hierarchical Clustering were performed to individuate differences in subgroups of samples. A Student'T-test was used to select the most deregulated features between GCT and OS and a false discovery rate procedure applied for multiple comparisons, setting the level of significance at 5%. The whole bioinformatics analysis was performed by MATLAB (The Mathworks Inc. Version 7.8).

### Target prediction

*In silico* putative target prediction of specific miRNAs and pathway analysis were conducted using miRWalk 2 (http://zmf.umm.uni-heidelberg.de/apps/zmf/mirwalk2/).

### Histological analysis

5μm-thick sections were trimmed from each formalin fixed paraffin embedded (FFPE) histological specimen (tab. 1, 3), re-hydrated and Haematoxylin/Eosin stained. Two expert pathologists independently assessed the GCT or OS diagnosis.

### U2OS and SAOS2 cell lines culture, treatment and transfection's conditions

U2OS and SAOS2 human osteosarcoma cell lines were cultured as monolayers at 37°C and 5% CO_2_ in DMEM (Invitrogen-Gibco, Carlsbad, CA, USA) supplemented with 10% non-heat inactivated FBS (fetal bovine serum). U2OS were treated with Cisplatin (TEVA Italia, Italy) at 1μg/ml and 5μg/ml. The dose-response curve indicates these doses have ineffective and 50% effective, respectively, after 24h (data not shown). For miR-181c over-expression, U2OS and SAOS2 cells, were transfected with the pCMV-miR-181c expression vector (miR-181c) and pCMV control vector (CNTR) (Origene Technologies, Rockville, MD, US) using Lipofectamine 2000 (Invitrogen-Gibco, Carlsbad, CA, US) following the manufacturer's instruction. While to inhibit miR-181c expression U2OS and SAOS2 cells were transfected with miRCURY LNA™ microRNA inhibitor (Exiqon, Vedbaek, Denmark) using Lipofectamine RNAiMAX (Invitrogen-Gibco, Carlsbad, CA, US) following the manufacturer's instruction. Cell growth was evaluated by luminescent assay using ATPlite™ Luminescence Assay System (PerkinElmer, Whaltman, MA, US).

### Cell viability assay

U2OS and SAOS2 cells where miR181c was either overexpressed or inhibited and their corresponding control were seeded in 96-well plate, 800 cells/well, four replicates per point. After 24h, cells were treated with the three drugs of the MAP chemotherapy regimen: Cisplatin (Teva Italia, srl) 1μg/ml; Doxorubicin (Ebewe Pharma, Austria) 0.01μg/ml, Methotrexate (Pfizer, New York, NY, US) 0.1μg/ml. The indicated concentrations lied below the IC_50_ values obtained by the single treatment dose-response curve of each drug (data not shown). After 24h, cell viability was evaluated with the ATPlite™ Luminescence Assay System following the manufacturer's instructions. Luminescence was evaluated with the EnSpire® Multimode Plate Reader (PerkinElmer, Whaltman, MA, USA) and the results were analyzed using the GraphPad Prism® software.

### Total cellular RNA extraction and RT-PCR

Total RNA was extracted from U2OS cells using Trizol Reagent (Invitrogen-Gibco, Carlsbad, CA, US) according to the manufacturer's instructions. RT-PCR quantification of miRNA expression was performed using TaqMan MiRNA Assays (Applied Biosystems) according to the manufacturer's protocol RNU48 was used as endogenous controls to standardize miRNA expression. All reactions were performed in duplicate.

### Western blot

U2OS cells were lysed in UREA buffer and protein concentrations were determined by colorimetric assay (Bio-Rad, Hercules, CA, US). Western blotting was performed using the following primary antibodies: mouse monoclonal anti-Cdc2 (Tyr-15) (Santa-Cruz Biotechnologies, Santa Cruz, CA, US); mouse monoclonal anti-Cyclin D1 (Invitrogen-Gibco, Carlsbad, CA, US); mouse monoclonal anti-tubulin (Santa-Cruz Biotechnologies, Santa Cruz, CA, US). A goat anti-mouse secondary antibody HRP-conjugated was used (Bio-Rad, Hercules, CA, US). Immuno-stained bands were detected by chemiluminescent method (Pierce, Rockford, IL, USA)

## SUPPLEMENTARY MATERIAL, FIGURES AND TABLES


