# Supplementary material for: miR-181c associates with tumor relapse of high grade osteosarcoma
Source: Oncotarget. 2015 Mar 12;6(16):13946–61. doi: 10.18632/oncotarget.3539 (PMC4546443; doi:10.18632/oncotarget.3539)
Supplement: Supplementary file 1 [file oncotarget-06-13946-s001.pdf]

miR-181c associates with tumor relapse of High Grade Osteosarcoma

Supplementary Material

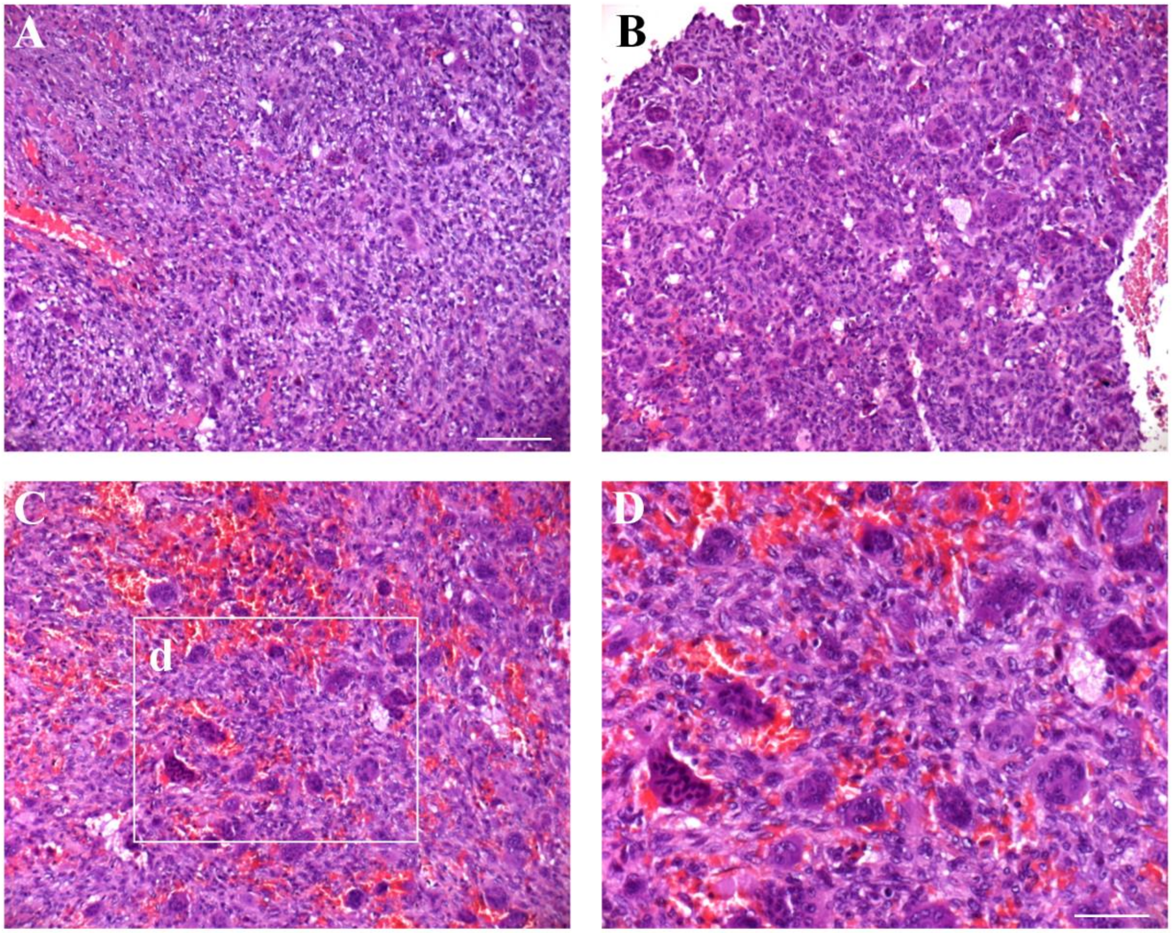

Supplementary fig. 1: A-C Three different fields from three independent FFPE specimens derived from the case report GCT and (D) a higher magnification field are reported. The tumor seems to be uniformly constituted by GCT characteristics, no traces of OS components can be recognized. A-C scale bar: 100µm; D scale bar: 50 µm.

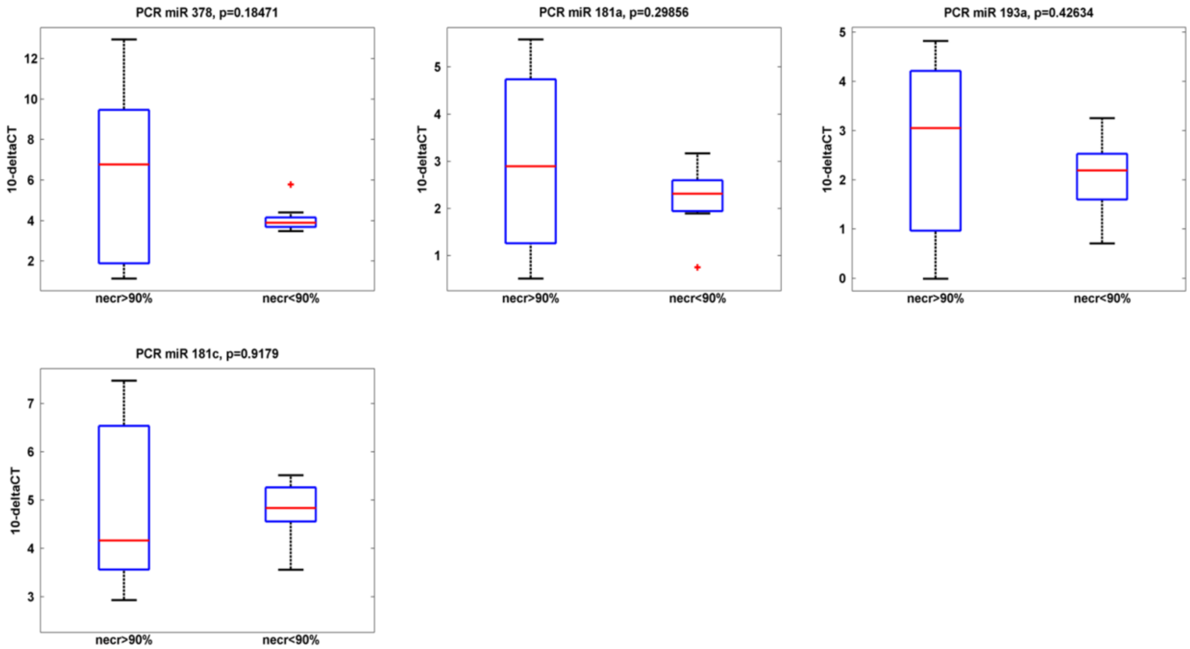

Supplementary fig. 2: miR-181a, miR193a, miR-181c and miR-378 expression levels in the sixteen OS biopsies from the Regina Elena National Cancer Institute casuistry. Box plots indicate that there is no correlation between the miRNAs expression levels and the necrosis induced by the chemotherapeutic treatment.

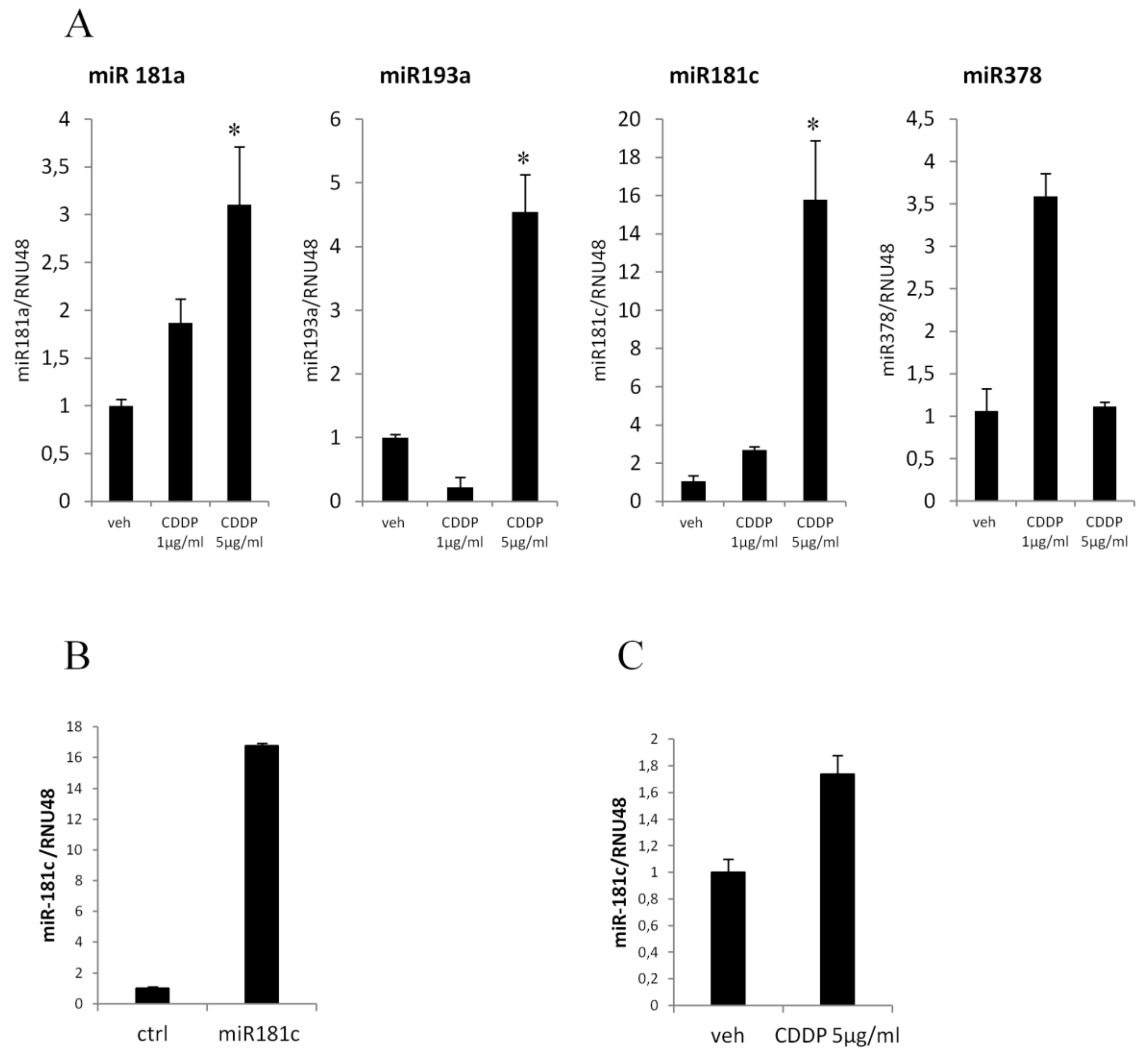

Supplementary fig. 3: miR-181a, miR-181c, miR193a and miR-378 expression levels in U2OS cells after 24h from CDDP treatment at the dosages of 1μg/ml and 5μg/ml. A statistically significant up-regulation in the expression of miR-181a, miR-181c, and miR193a is reported at the 5 μg/ml concentration (asterisks). B miR-181c expression levels in U2OS cells transfected with control and pCMV-miR-181c vectors, respectively. C. miR-181c expression levels in U2OS cells treated with 5μg/ml CDDP.

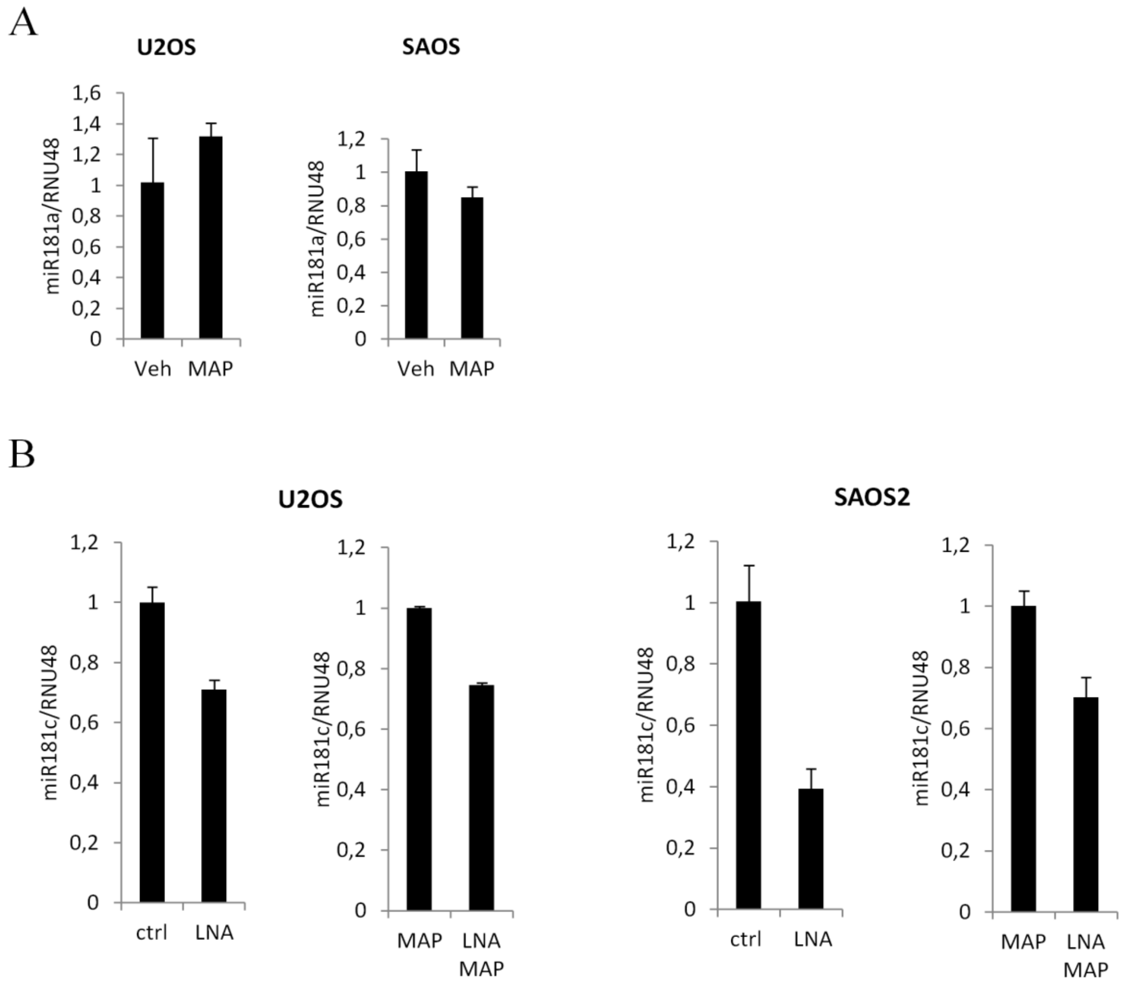

Supplementary fig. 4: A miR-181a levels of expression in U2OS and SAOS2 cell lines after MAP treatment. Differences in the levels of expression are not statistically significant. B miR-181c levels of expression in U2OS and SAOS2 transfected either with miRCURY LNA™ control (ctrl) or miRCURY LNA™ miR-181c inhibitor (LNA) with or without MAP treatment (MAP). Data are reported +/- SD.

Supplementary tab. 1: List of differently expressed miRNAs between GCT and OS (Patient A). FDR: False Discovery Rate.

miR	PVAL GCT VS OSTEO BETA	FDR	fold(osteo\GCT)
'hsa-miR-100'	0.008268487	0.00941947	-2.64
'hsa-miR-1202'	2.20E-06	1.08E-05	3.88
'hsa-miR-1207-5p'	4.23E-05	1.09E-04	3.81
'hsa-miR-1224-5p'	2.19E-04	4.05E-04	1.2
'hsa-miR-1225-5p'	2.59E-05	7.06E-05	3.66
'hsa-miR-1226*'	7.53E-03	8.78E-03	1.42
'hsa-miR-1246'	3.92E-05	0.00010375	3.57
'hsa-miR-125b'	4.15E-03	5.64E-03	-1.53
'hsa-miR-1260'	3.17E-02	2.45E-02	-1.83
'hsa-miR-1268'	1.27E-05	4.28E-05	3.02
'hsa-miR-1274b'	0.031681726	0.02463413	-1.58
'hsa-miR-1275'	8.75E-05	1.95E-04	1.41
'hsa-miR-1280'	0.013585063	0.0137211	-2.61
'hsa-miR-1290'	1.33E-06	7.65E-06	4.82
'hsa-miR-1323'	9.93E-08	2.43E-06	9.46
'hsa-miR-134'	1.54E-05	4.70E-05	3.47
'hsa-miR-140-3p'	9.43E-03	1.03E-02	-1.96
'hsa-miR-140-5p'	0.033845591	0.02512043	-1.86
'hsa-miR-149*'	1.38E-02	1.38E-02	2.96
'hsa-miR-150*'	7.35E-05	0.00016747	1.86
'hsa-miR-155'	0.003708845	0.00519086	1.29
'hsa-miR-181a'	0.000107072	0.00023311	-2.89
'hsa-miR-181a*'	0.002741932	0.00400941	-5.35
'hsa-miR-181b'	3.90E-04	6.71E-04	-2.14
'hsa-miR-181c'	0.01564304	0.01517395	-3.53
'hsa-miR-187*'	1.78E-06	9.18E-06	5.87
'hsa-miR-1915'	8.77E-06	3.18E-05	2.89
'hsa-miR-193a-5p'	0.039249314	0.02786454	-1.09
'hsa-miR-198'	0.030331558	0.02415953	3.04
'hsa-miR-199a-5p'	4.45E-02	3.09E-02	-1.65
'hsa-miR-218'	0.024705299	0.02141956	-4.74
'hsa-miR-22'	4.87E-02	3.20E-02	-1.02
'hsa-miR-222'	0.004395182	0.00589865	-2.91
'hsa-miR-30a'	0.045399218	0.03088764	-1.75
'hsa-miR-30c-2*'	0.046237473	0.03081596	2.49
'hsa-miR-320c'	0.000117033	0.00024396	1.7
'hsa-miR-373'	2.29E-06	1.07E-05	6.05
'hsa-miR-373*'	0.012779589	0.01346272	3.47
'hsa-miR-378'	0.006730387	0.00794439	-2.33
'hsa-miR-483-5p'	5.34E-05	0.00013422	2.37
'hsa-miR-494'	0.002363945	0.00361873	-2.43
'hsa-miR-497'	0.033535636	0.02508038	-1.93
'hsa-miR-498'	3.64E-07	4.46E-06	7.88
'hsa-miR-512-3p'	0.006549858	0.00782559	6.38
'hsa-miR-516a-5p'	1.45E-05	4.59E-05	1.77
'hsa-miR-516b'	2.67E-07	3.73E-06	8.48
'hsa-miR-517a'	0.004671903	0.0061853	9.28
'hsa-miR-517b'	1.96E-02	1.81E-02	8.79
'hsa-miR-518b'	1.02E-06	6.26E-06	6.79
'hsa-miR-518e'	1.60E-02	1.53E-02	7.24
'hsa-miR-519d'	3.88E-02	2.79E-02	7.36
'hsa-miR-520g'	0.038913343	0.02782767	7.32
'hsa-miR-522'	6.67E-08	6.53E-06	7.54
'hsa-miR-548g'	1.99E-02	1.82E-02	6.24
'hsa-miR-557'	0.000799858	0.00130605	3.04
'hsa-miR-572'	1.21E-04	2.48E-04	1.32
'hsa-miR-575'	0.000233758	0.0004241	1.53
'hsa-miR-583'	1.58E-05	4.68E-05	5
'hsa-miR-630'	6.88E-05	1.64E-04	2.97
'hsa-miR-638'	1.15E-04	2.46E-04	1.92
'hsa-miR-654-5p'	0.025950072	0.0221075	3.3
'hsa-miR-663'	7.68E-06	2.90E-05	4.41
'hsa-miR-718'	3.29E-02	2.48E-02	3.05
'hsa-miR-720'	0.000288103	0.0005132	-2.2
'hsa-miR-762'	5.72E-05	1.40E-04	1.76
'hsa-miR-765'	1.99E-03	3.10E-03	2.43
'hsa-miR-877'	8.20E-03	9.45E-03	6.07
'hsa-miR-939'	7.96E-04	1.32E-03	1.81
'hsa-miR-940'	3.13E-04	5.48E-04	2.22
'hsa-miR-99a'	1.30E-02	1.35E-02	-1.91

Supplementary tab. 2: Putative targeted genes identified by MiRWalk2 of miR-181a, miR-181c, miR-193a-5p and miR-378 involved in TGF-β and Wnt-pathways.

MicroRNA	Database	Gene Name	Pathway Name
hsa-miR-181a	KEGG	MAPK1, TGFBR2 , ACVR2B, SMAD7, ZFYVE16	TGF_BETA_SIGNALING_PATHWAY
hsa-miR-181c	KEGG	ACVR2B, MAPK1, SMAD7 TGFBR2, ZFYVE16	TGF_BETA_SIGNALING_PATHWAY
hsa-miR-193a-5p	KEGG	BMPR1B, ACVR1, INHBC	TGF_BETA_SIGNALING_PATHWAY
hsa-miR-378	KEGG	RBX1, BMP7, BMP8B, FST, GDF5 ID3, IFNG, RBL1, SMAD3, SMAD7, SP1	TGF_BETA_SIGNALING_PATHWAY
hsa-miR-181a	KEGG	WIF1, PPP3R1, BTRC, NFAT5, WNT16, WNT5B, CAMK2G, CSNK2A2, SFRP5, WNT11, CER1	WNT_SIGNALING_PATHWAY
hsa-miR-181c	KEGG	BTRC, CAMK2G, CER1 CSNK2A2, NFAT5, PPP2R5C PPP3R1, SFRP5, TCF7L2 WIF1, WNT11, WNT16, WNT5B	WNT_SIGNALING_PATHWAY
hsa-miR-193a-5p	KEGG	PLCB1, WNT3	WNT_SIGNALING_PATHWAY
hsa-miR-378	KEGG	RBX1, BMP7, BMP8B, FST GDF5, ID3, IFNG, RBL1 SMAD3, SMAD7, SP1	WNT_SIGNALING_PATHWAY
